# Peroxymonosulfate Activation by Rice-Husk-Derived Biochar (RBC) for the Degradation of Sulfamethoxazole: The Key Role of Hydroxyl Groups

**DOI:** 10.3390/ijms252111582

**Published:** 2024-10-29

**Authors:** Tong Liu, Chen-Xuan Li, Xing Chen, Yihan Chen, Kangping Cui, Qiang Wei

**Affiliations:** 1 School of Resources and Environmental Engineering, Hefei University of Technology, Hefei 230009, China; aimee777@ustc.edu.cn (T.L.); cxli@hfut.edu.cn (C.-X.L.); xingchen@hfut.edu.cn (X.C.); yhchen@hfut.edu.cn (Y.C.); 2 Key Laboratory of Nanominerals and Pollution Control of Higher Education Institutes, Hefei University of Technology, Hefei 230009, China; 3 CAS Key Laboratory of Urban Pollutant Conversion, Department of Environmental Science and Engineering, University of Science and Technology of China, Hefei 230026, China

**Keywords:** biochar, sulfamethoxazole, peroxymonosulfate, hydroxyl group

## Abstract

In this work, rice-husk-derived biochar (RBC) was synthesized by using simple one-step pyrolysis strategies and served as catalysts to activate peroxymonosulfate (PMS) for degrading sulfamethoxazole (SMX). When the annealing temperature (T) = 800 °C, RBC_800_ exhibits the typical hardwood structure with several micropores and mesoporous. Furthermore, RBC_800_ obtains more defect sites than RBC_600_, RBC_700_, and RBC_900_. In the RBC_800_/PMS system, the removal rate of the SMX reached 92.0% under optimal conditions. The kinetic reaction rate constant (*k*_obs_) of the RBC_800_/PMS system was 0.009 min^−1^, which was about 1.50, 1.28, and 4.50 times that of the RBC_600_/PMS (*k*_obs_ = 0.006 min^−1^), RBC_700_/PMS (*k*_obs_ = 0.007 min^−1^), and RBC_900_/PMS (*k*_obs_ = 0.002 min^−1^) systems, respectively. In the RBC_800_/PMS system, sulfate radical (SO_4_^•−^) is the main active species. Compared with other active sites, the hydroxyl group (C-OH) on the surface of RBC_800_ interacts more strongly with PMS, which is more likely to promote the stretching of the O-O bond of the PMS, thus breaking into the activated state and significantly reducing the activation energy required for reaction. The degradation intermediates of SMX were speculated, and the toxicity analysis was conducted. Generally, this work reveals in depth the interaction between reactive sites of biochar-based catalysts and PMS at the molecular level.

## 1. Introduction

Recently, pharmaceuticals and personal care products (PPCPs) have been widely adopted to treat a variety of human and animal diseases [1]. Among them, sulfamethoxazole (SMX) as a typical PPCPs can effectively prevent coccidiosis, diarrhea, gastroenteritis, and other bacterial diseases [2]. SMX is first consumed by humans or animals and enters the wastewater treatment system with excreta. However, most conventional treatment processes cannot degrade SMX effectively, so residual SMX enters the natural water environment with treated municipal wastewater [3]. Residual SMX in aquatic environments may increase the resistance of pathogenic microorganisms, causing negative effects on aquatic life [4]. Therefore, it is important to develop an effective method for eliminating residual SMX in the aquatic environment.

The hydroxyl radical-based advanced oxidation processes (HR-AOPs) are the effective method for degrading emerging contaminants, and their mechanisms are largely dependent on the hydroxyl radical (^•^OH) [5]. However, HR-AOPs also have many shortcomings, such as the difficulty of transporting and storing hydrogen peroxide (H_2_O_2_), a narrow pH application range, and higher energy consumption [6]. Recently, persulfate-based advanced oxidation processes (PS-AOPs) have been favored by researchers due to higher oxidizability and wider applicability under the environments (pH = 2.0–8.0) [5]. There are lots of methods for peroxydisulfate (PDS) and peroxymonosulfate (PMS) activation to generate reactive oxygen species (ROSs), such as ultraviolet irradiation, heat, and transition metals [7]. However, these activation methods often have problems such as extensive energy consumption, high cost, metal leaching, etc., which limits their application prospects in actual wastewater [8].

Biochar (BC) has become a research hotspot recently [9]. Biomass feedstock, such as sludge, corncob, wood chips and shrimp shell, is rich and cheap [10]. Besides, BC has abundant functional groups as well as well-developed porous structure, which obtains significant advantages for the synthesis of carbonaceous catalysts [11]. Qi et al. reported that the *Enteromorpha* based graphene-like biochar (EGB) was synthesized as a PDS activator for the SMX removal [12]. Liu et al. reported that boron-doped graphitic porous biochar (B-KBC) was synthesized and used as catalysts to activate PDS for removing SMX [13]. Besides, Zhao et al. systematically reported the research progress of the PMS activation by biochar-based catalysts [11]. Although some studies have been carried out on the degradation of micropollutants via activating PS by pristine BC, the interaction mechanism between surface active sites of BC and PS is still unclear. In addition, the pyrolysis temperature (T) largely determines the structure and physicochemical properties of catalysts, while the specific surface area (SSAs), micropore structure and graphitization degree of BC will affect the removal rate of organic micropollutants in PS-AOPs [11]. Herein, the structure-activity relationship between the physicochemical properties of catalysts and the catalytic activity needs to be further revealed.

Herein, raw biochar (RBC_T_) derived from rice husk was prepared as a PMS activator for the elimination of SMX. The difference of physicochemical properties of RBC_T_ at different pyrolysis temperatures was investigated, and the structure-activity relationship between physicochemical properties and catalytic activity was established. Then, the ROSs in the RBC_800_/PMS system were identified. The degradation pathway of SMX was predicted, and the interaction mechanism between active sites and PMS was revealed by density functional theory (DFT) calculation. The study proposes a deep understanding of PMS activation mechanism and demonstrates great potential of the biochar-based catalysts toward sewage treatment.

## 2. Results and Discussion

### 2.1. Characterization of RBC_800_

**Figure 1:**
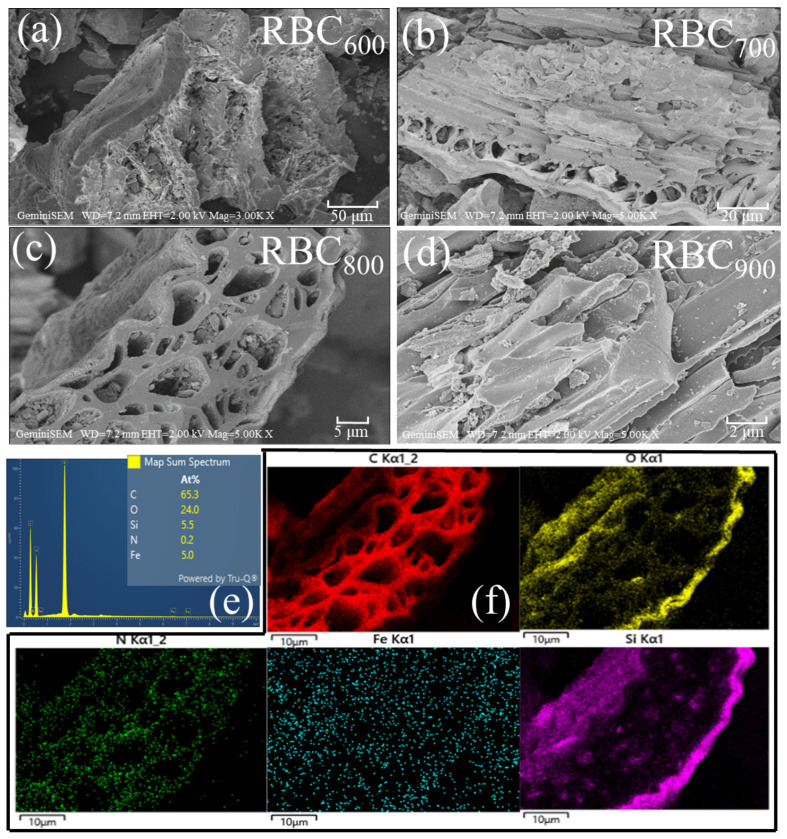
(**a**–**d**) SEM images of RBC_T_; (**e**) EDS elemental content, and (**f**) element mappings of RBC_800_.

The morphology of RBC_T_ were observed by scanning electron microscopy (SEM). As displayed in Figure 1a–d, RBC_600_ shows the clumpy structure and incomplete porous structure. When the pyrolysis temperature is 700 °C, RBC_700_ shows a plate structure, and the pore structure has initially developed. RBC_800_ derived from rice husks exhibits a typical hardwood structure, with lots of micropores and mesoporous. When the pyrolysis temperature is 900 °C, the carbon network collapses. It can be seen that the porous structure of RBC is relatively developed when the pyrolysis temperature is 800 °C, which is conducive to the catalytic oxidation process. However, excessive pyrolysis temperature (T ≥ 900 °C) may destroy the well-developed porous structure.

As depicted in Figure 1e,f, energy-dispersive X-ray spectroscopy (EDS) analyzed the elemental content of RBC_800_, in which C content accounted for 65.3%, O content accounted for 24.0%, Si content accounted for 5.5%, N content accounted for 0.2% and Fe content accounted for 5.0%. Besides, Fe element is uniformly distributed on RBC_800_ surface. These results indicate that rice husk-derived RBC_800_ naturally contains a small amount of Fe element without additional iron source.

**Figure 2:**
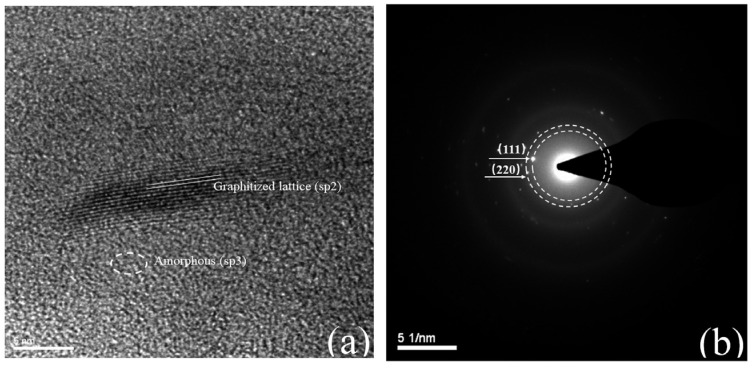
(**a**) HRTEM, and (**b**) SAED pattern of RBC_800_.

Furthermore, the ultrastructure of crystal lattice of RBC_800_ was observed in the high-resolution transmission electron microscope (HRTEM) and selected area electron diffraction (SAED) pattern, as depicted in Figure 2a,b. RBC_800_ is composed of graphitized (sp^2^C) and disordered carbon (sp^3^C) structures, and the degree of graphitization and disordered of the catalyst need to be further determined by Raman spectroscopy. The Miller indices of (111) and (220) is consistent with the X-ray diffraction (XRD) results.

**Figure 3:**
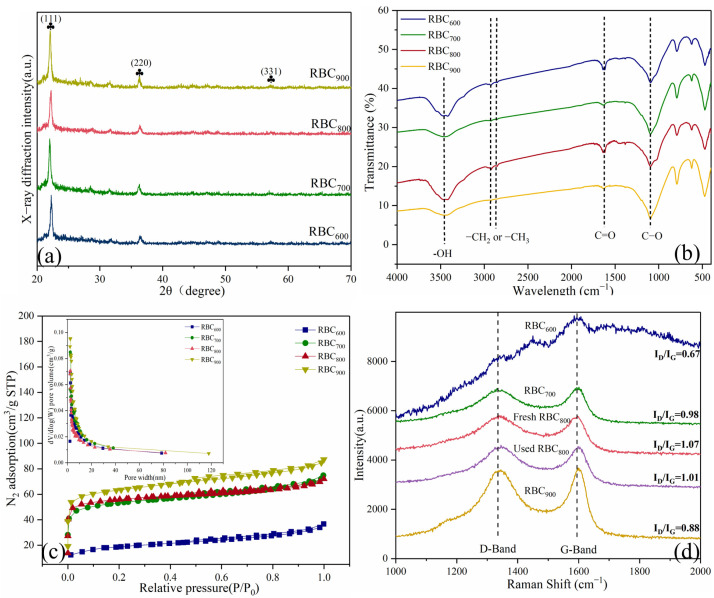
(**a**) XRD patterns, (**b**) FTIR spectra, (**c**) Nitrogen adsorption–desorption isotherms and pore structure (the inset), and (**d**) Raman spectra of RBC_T_.

XRD patterns of RBC_T_ are depicted in Figure 3a. Obviously, the pronounced diffraction peak at 21.60° was attributed to the (111) plane of SiO_2_ [14]. Two other weak peaks at 35.6° and 56.2°, corresponding to the (220) and (331) planes, agree well with the crystalline phase of calcite (JCPDS 27-0605).

FT-IR spectra show the functional groups of RBC_T_ (Figure 3b). The peaks at 1250 cm^−1^, 1622 cm^−1^, and 3419 cm^−1^ are respectively attributed to the stretching vibration of C-O, C=O and -OH groups [15]. Xin et al. reported that the content of functional groups can be qualitatively judged by the absorption peak intensity [6]. These results show that the annealing temperature has a crucial effect on the formation of oxygen-containing functional groups. The content of C=O and -OH groups on RBC_800_ surface is significantly higher than that on RBC_600_, RBC_700_ and RBC_900_, while the C=O and -OH groups on the surface of catalyst can promote electron transfer processes and activate PMS to generate ROSs [16].

N_2_ adsorption-desorption isotherms (Figure 3c) indicate the specific surface areas (SSAs) of RBC_800_ (194.86 m^2^⋅g^−1^) are larger than that of RBC_600_ (65.00 m^2^⋅g^−1^), RBC_700_ (124.11 m^2^⋅g^−1^) and RBC_900_ (102.71 m^2^⋅g^−1^) (Table S1). The appearance of both D-band and G-band of RBC_T_ reveals the co-presence of disordered and crystalline graphite structures [17] (Figure 3d). Furthermore, the ratio of I_D_/I_G_ is the key parameter to indicate the defective degree of RBC_T_ [18,19]. The I_D_/I_G_ value was obtained by calculating the intensity of ration of D peak to G peak [13]. The I_D_/I_G_ is 0.67, 0.98, 1.07, and 0.88 for RBC_600_, RBC_700_, RBC_800_, and RBC_900_, respectively. The result demonstrates that RBC_800_ has obtained abundant defects (vacancy and edge defects) during pyrolysis, which is conducive to catalytic oxidation [20].

### 2.2. Catalytic Oxidation of SMX

**Figure 4:**
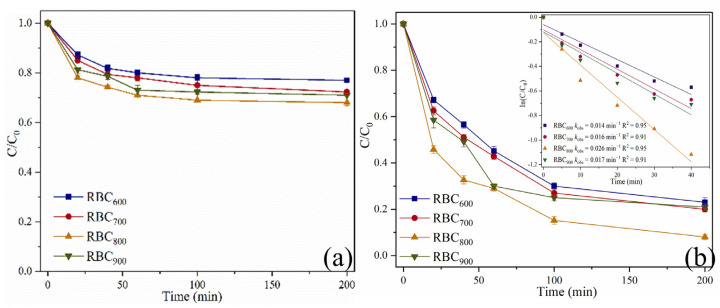
The adsorption rate (**a**) and degradation rate (**b**) of SMX in various reaction systems. Reaction conditions: [SMX]_0_ = 10.0 mg/L; [RBC_T_] = 0.4 g/L; [PMS]_0_ = 0.6 mM; pH = 7.0; Reaction temperature = 25 °C.

The adsorption process accords with Langmuir model, suggesting the monolayer adsorption of SMX on RBC_800_ surface (Figure S1). In the pure RBC_T_ system, 23.0%, 27.7%, 32.0%, and 29.0% of SMX could be adsorbed onto RBC_600_, RBC_700_, RBC_800_, and RBC_900_ within 200 min, respectively (Figure 4a). RBC_800_ has higher SMX adsorption capacities, due to its larger SSAs and developed porous structure [21].

In the RBC800/PMS system, the degradation efficiency of SMX was 67.3% within 40 min, indicating that BC can activate PMS to a certain extent [22]. In the presence of PMS, the removal rate of RBC_600_, RBC_700_, RBC_800_, and RBC_900_ within 200 min was 77.0%, 80.0%, 92.0%, and 79.0%, respectively (Figure 4b). The removal process of SMX followed a first-order kinetics behavior, and *k*_obs_ is the reaction rate constant. The *k*_obs_ of RBC_800_/PMS system was 0.009 min^−1^, being about 1.50, 1.28, and 4.50 times that of RBC_600_/PMS, RBC_700_/PMS, and RBC_900_/PMS systems, respectively (Table S2).

It indicated that the annealing temperature (T) can affect the catalytic performances of catalysts. Within a certain range (T < 900 °C), the increase of pyrolysis temperature is conducive to the development of micropores and promotes the conversion of the organic phase with poor crystallinity into graphitic carbon structure, thus enhancing the catalytic performance of catalysts [23]. However, when the pyrolysis temperature is too high (T = 900 °C), the collapse of the carbon skeleton causes a partial loss of the defects, leading to a decrease in the catalytic activity of RBC_T_ [21]. The PPCPs degradation rates in different systems were studied in Table S3. Besides, the effect of various factors on SMX degra-dation were investigated (Figure S2). The point of zero charge (pH_pzc_) of RBC_800_ was displayed in Figure S3. To explore the catalytic performance of RBC_800_ in depth, the residual PMS concentration was detected by the ABTs colorimetric method. The decomposition rate of PMS in the RBC_800_/PMS system was 74.9% within 200 min (Figure S4). It was suggested that the favorable degradation rate of SMX in the RBC_800_/PMS system could be due to the rapid decomposition of PMS.

Co-existing ions can affect the SMX degradation by interacting with ROSs [24]. As shown in Figure S5, typical anions (HCO_3_^−^, Cl^−^, H_2_PO_4_^−^) and humic acid (HA) may restrain the SMX elimination. When 5.0 mM and 10.0 mM Cl^−^ were present in the RBC_800_/PMS system, SMX elimination rate decreased from 92.0% to 73.0% and 58.0%, respectively, within 200 min. The *k*_obs_ decreased from 0.009 min^−1^ to 0.003 min^−1^ and 0.002 min^−1^, which was due to the generation of Cl^•^, Cl_2_, and HOCl (Equations (1)–(5)) [25]. When increasing the H_2_PO_4_^−^ concentration to 5.0 mM and 10.0 mM, the SMX removal rate decreased to 70.0% and 57.7%, respectively. Similarly, when HCO_3_^−^ concentration was increased from 0 to 5.0 mM and 10.0 mM, the SMX elimination rate decreased from 92.0% to 66.3% and 60.0%, respectively. These results reveal that H_2_PO_4_^−^ and HCO_3_^−^ possess a quenching effect on reactive radicals (Equations (6)–(9)) [26]. When 5.0 mg⋅L^−1^ and 10.0 mg⋅L^−1^ of HA was added into the RBC_800_/PMS system, *k*_obs_ decreased from 0.009 min^−1^ to 0.003 min^−1^ and 0.002 min^−1^, respectively. The π-π stacking effect of HA can lead to competing adsorption between HA and PMS, thus hinder the production of active species [27].

(1) $$Cl^{-}+SO_{4}^{\cdot -}\to SO_{4}^{2-}+{Cl}^{\cdot }$$

(2) $$HSO_{5}^{-}+Cl^{-}\to SO_{4}^{2-}+HOCl$$

(3) $$HSO_{5}^{-}+2Cl^{-}+H^{+}\to SO_{4}^{2-}+{Cl}_{2}+H_{2}O$$

(4) $$Cl^{-}+\cdot OH\to HOCl^{\cdot -}$$

(5) $$HOCl^{\cdot -}+H^{+}\to {Cl}^{\cdot }+H_{2}O$$

(6) $$OH+H_{2}PO_{4}^{-}\to H_{2}PO_{4}^{\cdot }+OH^{-}$$

(7) $$SO_{4}^{\cdot -}+H_{2}PO_{4}^{-}\to H_{2}PO_{4}^{\cdot }+SO_{4}^{2-}$$

(8) $$HCO_{3}^{-}{+}^{\cdot }OH\to H_{2}O+CO_{3}^{2-}$$

(9) $$HCO_{3}^{-}+SO_{4}^{\cdot -}\to SO_{4}^{2-}+HCO_{3}^{\cdot }$$

To study the universal applicability of RBC_800_ in real waterbody, more tests were conducted in various water matrices. The characteristics of different water matrices were listed in Table S4. The removal rate of SMX in tap water (79.0%) was lower than that obtained for deionized water (92.0%) (Figure S6a). Besides, the elimination rate decreased in river water, which might be due to the presence of certain level of ions in the river water [28].

To explore the reusability of RBC_800_, five consecutive degradation tests were performed (Figure S6b). After four cycles, the elimination rate of SMX within 200 min decreased from 92.0% to 66.3%. This could be due to the sedimentation of intermediates on the surface of RBC_800_, which occupied the defective sites for activating PMS [15]. To recover the porous structure of passivated RBC_800_, a thermal treatment (annealing at 450 °C under N_2_ flow for 2 h) was applied. The result showed that the catalytic activity was partially recovered with 74.0% within 200 min in the RBC_800_/PMS system.

To further explore the wide applicability of RBC_800_, the degradation experiments of different pollutants were carried out (Figure S6c). In the RBC_800_/PMS system, the degradation rates of CEX, CIP, CMP and TCS within 200 min were 87.3%, 91.1%, 90.2% and 89.6%, respectively, and the mineralization rates were 67.4%, 72.3%, 73.7% and 76.1%, respectively. These results showed that the admirable universality of RBC_800_ as an efficient PMS activator to remove a broad array of typical PPCPs. As displayed in Figure S6d, the RBC_800_/PMS system had a satisfactory mineralization performance on SMX with a TOC elimination rate of 79.0% within 200 min.

### 2.3. Mechanism Discussion

#### 2.3.1. Identification of ROSs

**Figure 5:**
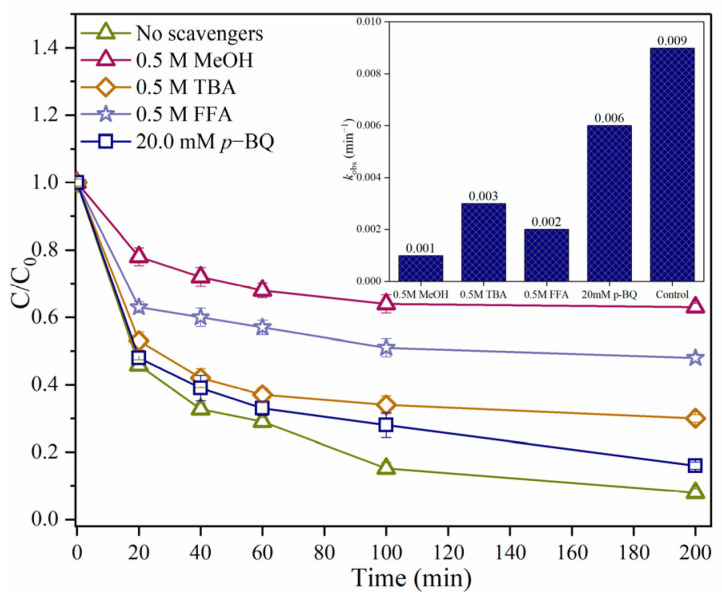
Effects of ROSs scavengers on the SMX degradation in the RBC_800_/PMS system. Reaction conditions: [SMX]_0_ = 10.0 mg/L; [RBC_800_] = 0.4 g/L; [PMS]_0_ = 0.6 mM; pH = 7.0; MeOH = 0.5 M; TBA = 0.5 M; FFA = 0.5 M; *p*-BQ = 20.0 mM; Reaction temperature = 25 °C.

Quenching tests were conducted to first to determine the dominant ROSs responsible for SMX degradation process [13,29]. Methanol (MeOH) was regarded as a scavenger of ^•^OH ((1.6–7.7) × 10^7^ M^−1^s^−1^) and SO_4_^•−^ ((1.2–2.8) × 10^7^ M^−1^s^−1^) [30]. In contrast, *Tert*-butanol (TBA) was used as a scavenger to quench ^•^OH (6.0 × 10^8^ M^−1^s^−1^) [31]. *p*-benzoquinone (*p*-BQ) was used to inhibit O_2_^•−^ (9.6 × 10^8^ M^−1^s^−1^) [24]. The SMX elimination rates decreased from 92.0% to 37.0%, 70.0%, 84.0%, and 52.0% within 200 min after adding MeOH (0.5 M), TBA (0.5 M), *p*-BQ (20.0 mM), and FFA (0.5 M), respectively (Figure 5). The results showed that MeOH had an obvious inhibitory effect on SMX degradation in the RBC_800_/PMS system, while TBA had a mild inhibitory effect on SMX degradation. Therefore, SO_4_^•−^ may be the main ROSs, leading the degradation process of SMX. In addition, O_2_^•−^ had little effect on the degradation of SMX, while ^•^OH and ^1^O_2_ were also involved in the degradation of SMX.

**Figure 6:**
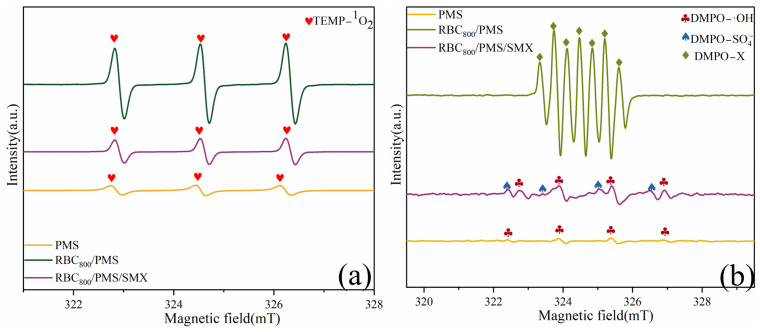
ESR signals of (**a**) TEMP-^1^O_2_ and (**b**) DMPO-^•^OH and DMPO-SO_4_^•−^. (Conditions: [SMX]_0_ = 10.0 mg/L; [RBC_800_]_0_ = 0.4 g/L; [PMS]_0_ = 0.6 mM; pH = 7.0; Reaction temperature = 25 °C; [TEMP] = [DMPO] = 10.0 mM).

ESR was used to monitor the presence of main active species in the RBC_800_/PMS system using 2,2,6,6-tetramethyl-4-piperidinol (TEMP) and 5,5-dimethyl-1-pyrroline N-oxide (DMPO) as spin-trapping agents [32]. When RBC_800_, PMS and TEMP were added to the system, a typical triplet signal with the intensity ratio of 1:1:1 verified the existence of ^1^O_2_. The self-decomposition of PMS could produce a small amount of ^1^O_2_ [33] (Figure 6a). After adding SMX to the RBC_800_/PMS system, the peak intensity of TEMP-^1^O_2_ was significantly weakened, which indicated that ^1^O_2_ played a role in the degradation process of SMX. A spectrum with seven main peaks belonging to DMPO-X was observed in the RBC_800_/PMS system, suggesting that RBC_800_ activated PMS to produce SO_4_^•−^ and ^•^OH (Figure 6b). After adding SMX to the RBC_800_/PMS system, DMPO-^•^OH and DMPO-SO_4_^•−^ signals were significantly weakened, which indicated that ^•^OH and SO_4_^•−^ played a vital role in the degradation process of SMX. According to the quenching experiment and ESR test results, ^1^O_2_, SO_4_^•−^ and ^•^OH were produced in the RBC_800_/PMS system, and SO_4_^•−^ played a dominant role in the removal of SMX.

#### 2.3.2. Reaction Mechanism

**Figure 7:**
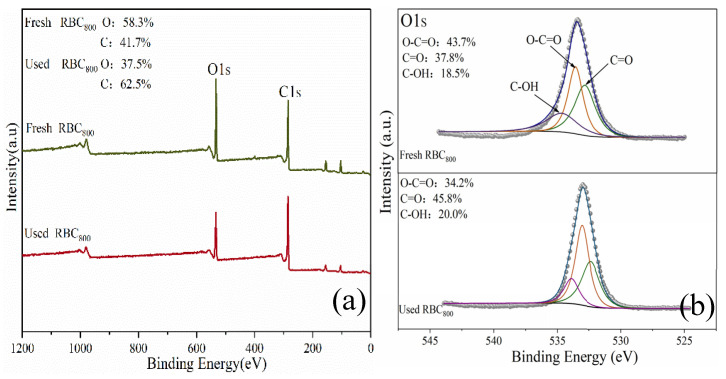
XPS spectra of full-range survey (**a**), and O 1s (**b**) of RBC_800_.

PMS activation mechanism was further investigated in this work. First, ^•^OH and SO_4_^•−^ generated from the destruction of the O-O bond of the PMS by free-flowing π electrons on the *sp*^2^ hybrid carbon of the RBC_800_ (Equations (10) and (11)). Secondly, ^1^O_2_ could be produced by the self-decomposition of PMS (Equations (12) and (13)) [33]. According to FT-IR and XPS results, the surface of RBC_800_ contained C-OH, COOH, C=O and other oxygen-containing functional groups, which could be used as the active center of RBC_800_. Two characteristic peaks attributed to C 1s and O 1s were observed on the full XPS spectra with binding energies at 285.1 eV and 531.1 eV, respectively (Figure 7a). The O1s XPS spectra can be decomposed into three components (Figure 7b). The peak at 531.8 eV and 533.3 eV could be assigned to C=O and COOH, respectively. A peak centered at 534.1 eV corresponds to C-OH. After the reaction, the content of COOH decreased from 43.7% to 34.2%, suggesting that COOH, as the active site in the catalytic reaction, activated PMS to produce SO_4_^•−^. In addition, C-OH on the surface of RBC_800_ can also activate PMS to produce SO_4_^•−^, and the conversion between SO_4_^•−^ and ^•^OH can be flexible (Equations (14)–(17)) [16]. Due to electrostatic attraction and the interaction between electron donors and acceptors, the adsorption of RBC_800_ facilitated the uniform distribution of SMX molecules on the surface of the carbon matrix, promoting contact with ROSs [34].

(10) $$\pi –electrons+HSO_{5}^{-}\to SO_{4}^{\cdot -}+OH^{-}$$

(11) $$\pi –electrons+HSO_{5}^{-}\to^{\cdot }OH+SO_{4}^{2-}$$

(12) $$HSO_{5}^{-}\to SO_{5}^{2-}+H^{+}$$

(13) $$HSO_{5}^{-}+SO_{5}^{2-}\to SO_{4}^{2-}+HSO_{4}^{-}+1O_{2}$$

(14) $$BC–OOH+HSO_{5}^{-}\to SO_{4}^{\cdot -}+BC–OO^{\cdot }+H_{2}O$$

(15) $$BC–OH+HSO_{5}^{-}\to SO_{4}^{\cdot -}+BC–O^{\cdot }+H_{2}O$$

(16) $$SO_{4}^{\cdot -}+H_{2}O\to H^{+}+SO_{4}^{2-}{+}^{\cdot }OH$$

(17) $${\,}^{\cdot }OH/SO_{4}^{\cdot -}/1O_{2}+SMX\to intermediates\to degraded\;products+CO_{2}+H_{2}O$$

Based on the above analysis results, *sp*^2^ hybrid carbon on the RBC_800_ surface and oxygen-containing functional groups such as COOH, C-OH and C=O could be used as the active sites, but the interaction mechanism between PMS and these active sites has not been deeply revealed. Therefore, density functional theory (DFT) calculations were adopted to further explore the activation mechanism of PMS on the surface of RBC_800_.

**Figure 8:**
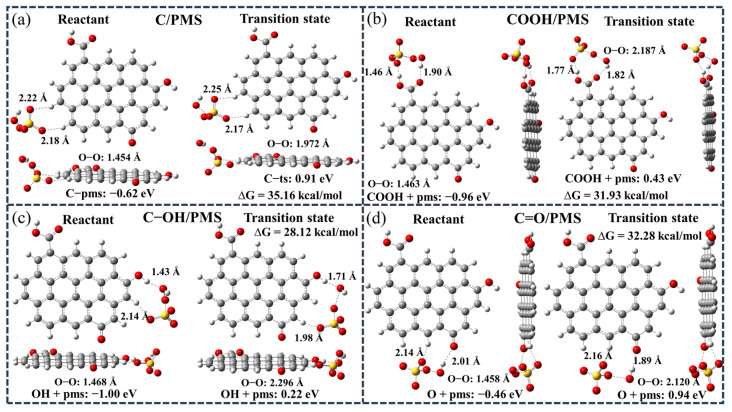
The optimization structures of PMS adsorption on different sites and the corresponding transition state. (**a**) C/PMS, (**b**) COOH/PMS, (**c**) C-OH/PMS and (**d**) C=O/PMS.

The adsorption processes of PMS on *sp*^2^ hybrid carbon network (C/PMS), carbon network edge containing COOH functional group (COOH/PMS), carbon network edge containing C-OH functional group (C-OH/PMS) and carbon network edge containing C=O functional group (C=O/PMS) were studied. The top and side views of all the optimized configurations are shown in Figure 8. The shortest distance (D) between PMS and *sp*^2^ hybrid carbon network, COOH, C-OH and C=O were 2.18 Å, 1.46 Å, 1.43 Å and 2.01 Å, respectively (Table S5). Compared with other configurations, the C-OH/PMS configuration has the smallest D value, suggesting that there may be a strong interaction between C-OH and PMS.

The essence of PMS activation is the breaking of O-O bond. The O-O bond length (*l*_o-o_) of PMS at *sp*^2^ hybrid carbon network, COOH, C-OH and C=O is 1.454 Å, 1.464 Å, 1.468 Å and 1.458 Å, respectively. These results indicate that oxygen-containing functional groups can act as reactive sites and promote the stretching of O-O bond of PMS. The *l*_o-o_ of PMS is the longest at C-OH, suggesting that the C-OH functional group could largely promote the stretching of the O-O bond of PMS, thus breaking into the activated state.

**Figure 9:**
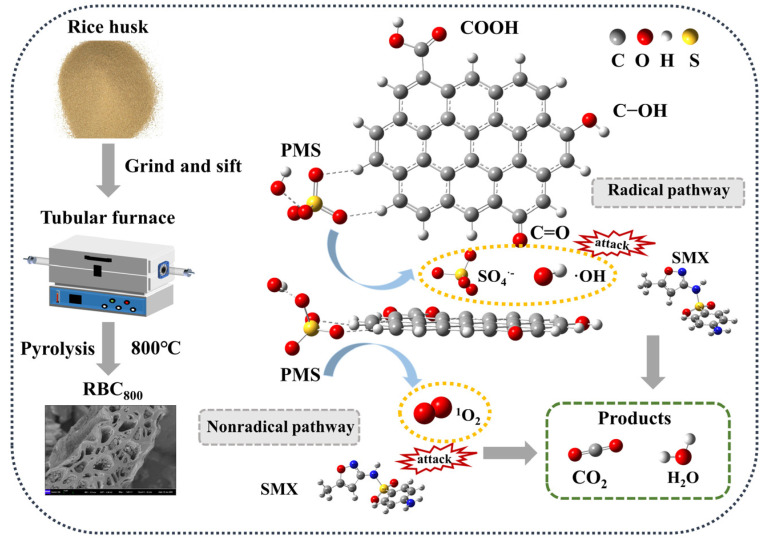
Proposed mechanism of SMX degradation in the RBC_800_/PMS system.

The energy barrier required for the reaction is calculated by optimizing the reactants and transition states. The energy barriers corresponding to the C/PMS, COOH/PMS, C-OH/PMS and C=O/PMS configurations are 35.16 kcal/mol, 31.93 kcal/mol, 28.12 kcal/mol and 32.28 kcal/mol, respectively. Obviously, the transition state energy barrier corresponding to the C-OH/PMS configuration is low, indicating that the C-OH functional group can significantly reduce the activation energy required for the reaction, and the catalytic activity of this active site is high. Based on the above discussion, Figure 9 displays the degradation mechanism of SMX in the RBC_800_/PMS system.

### 2.4. Degradation Pathways of SMX

The intermediates of SMX degradation were investigated by UPLC-TOF/MS (Figures S7 and S8 and Table S6). In pathway I, under the attack of ^•^OH, electrophilic addition reaction occurred on the C atom of the isoxazole ring of SMX to produce product I (m/z = 288) [2]. In addition, SO_4_^•−^ may also attack the olefin double bond on the isoxazole ring of the SMX molecule and form product I [35]. Subsequently, ^•^OH further attacks the S-N bond, transforming product I into product II (m/z = 133) and product III (m/z = 190). Surface charge distribution of SMX manifests that negatively charge regions are concentrated around the O and N atoms (Figure S9). Via the attack of ROSs, the N-O bond on the isoxazole ring breaks to produce product IV (m/z = 117), which is eventually mineralized into CO_2_ and H_2_O. In pathway II, under the attack of ^•^OH, SMX molecules first undergo nitration reaction to produce product VI (m/z = 284), then break S-C bond on SMX molecules and undergo hydroxylation reaction to produce product VII (m/z = 143), and then undergo benzene ring opening reaction and transform into small molecular organic matter, and eventually mineralized into CO_2_ and H_2_O.

### 2.5. Toxicity Assessment

**Figure 10:**
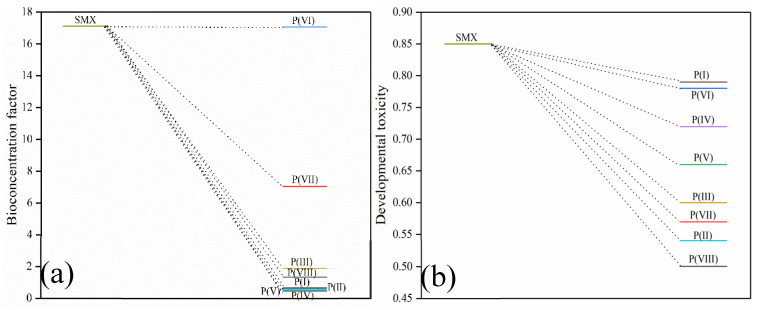
Bioaccumulation factor (**a**), and developmental toxicity (**b**) of SMX and its degradation byproducts.

The bioaccumulation factor and developmental toxicity of SMX as well as its intermediate products were estimated by Toxicity Estimation Software Tool (T.E.S.T) (5.1.1.0). T.E.S.T has a simple interface and easy operation, and the software is equipped with a guide, which is easy to use. Users can draw and load the structure of chemical substances by CAS number, SMILES code, substance name, InChi code, DTXSID or manually, then select the prediction end point and method, and change the output path of the result. The software automatically generates a result report after the prediction. The bioaccumulation factor of SMX was 17.11 mg/L. The bioaccumulation factor of P(I), P(II), P(III), P(IV), P(V), P(VI), P(VII) and P(VIII) were 0.67, 0.59, 1.89, 0.44, 0.49, 17.06, 7.04 and 1.34 mg/L, respectively (Figure 10a). The developmental toxicity of SMX was 0.85 mg/L. The developmental toxicity of P(I), P(II), P(III), P(IV), P(V), P(VI), P(VII) and P(VIII) were 0.79, 0.54, 0.60, 0.72, 0.66, 0.78, 0.57 and 0.50 mg/L, respectively (Figure 10b). The bioaccumulation factor and developmental toxicity of all intermediates was lower than that of SMX. According to the results of toxicity analysis, the comprehensive environmental risk of SMX was decreased in the RBC_800_/PMS system.

## 3. Materials and Methods

### 3.1. Preparation of Catalysts

Firstly, Rice husk was washed, oven-dried at 100 °C for 12 h, ground, and then passed through a 100-mesh sieve to acquire thin powders for further use. BC was prepared by pyrolysis at a constant calcination temperature under N_2_ atmosphere (5 °C/min of heating rate) for 2 h. The resulting powers are further ground, sieved and then stored in a ziplock bag. To further remove ash, soluble salts and other impurities from biochar, and increase the content of acidic functional groups on the BC surface, 100.0 g of the collected sample was added to 300.0 mL of 0.1 M hydrochloric acid (HCl, 36.0–38.0%) solution. The mixture was stirred for 24 h, then filtered, and the biochar is washed with ultra-pure water for several times to stabilize its pH to 6.0. The biochar was dried at 100–105 °C for 12 h after cleaning, and the resulting composities were denoted as RBC_T_ (T = 600, 700, 800, 900 °C).

### 3.2. Reaction Procedures

Degradation tests were conducted in 50 mL vials to investigate the catalytic activity of RBC_T_. The range of catalyst dosing was 0.2–1.0 g/L, the pH was 3.0–11.0, the PMS concentration was 0.2–1.0 mM, and the reaction temperature was set to 25 °C. The batch experiments were carried out at 150 rpm, and 1.0 mL reaction solution was taken periodically and filtered through a 0.22 μm filter. The redundant reaction was restrained by adding 1 mL Na_2_S_2_O_3_ (0.2 M). All tests were conducted in triplicate, and the final values were averaged.

## 4. Conclusions

Raw biochar (RBC_T_) was prepared by single one-step pyrolysis strategies using green and cheap rice husk biomass as precursor. The structure-activity relationship between physicochemical properties of RBC_T_ and catalytic activity is discussed in detail. The pyrolysis temperature (T) has a crucial influence on the morphology and structural characteristics of RBC_T_, which determines the catalytic performance of RBC_T_. When T < 800 °C, the porous structure of RBC_T_ is not fully developed, and when T > 800 °C, the carbon skeleton of RBC_T_ collapses. When T = 800 °C, RBC_800_ exhibits a typical hardwood structure with micropores and mesoporous. In addition, RBC_800_ obtains more defect sites than RBC_600_, RBC_700_, and RBC_900_. Therefore, under optimal conditions, the elimination rate of SMX in the RBC_800_/PMS system within 200 min was 92.0%, *k*_obs_ is 0.009 min^−1^, which is 1.8, 1.6 and 1.5 times that of the RBC_600_/PMS, RBC_700_/PMS and RBC_900_/PMS systems. Compared with similar reports, rice husk-derived biochar has obtained higher degradation performance [36]. Radical quenching, ESR and XPS analysis manifested that the main ROSs in the RBC_800_/PMS system were measured to be SO_4_^•−^. According to the DFT calculation results, compared with other active sites, the C-OH functional group on the surface of RBC_800_ interacts more strongly with PMS, which is more likely to promote the stretching of the O-O bond of PMS, thus breaking into the activated state and significantly reducing the activation energy required for reaction. This work reveals in depth the interaction mechanism between active sites of biochar-based catalysts and PMS at the molecular level. Besides, the structure-activity relationship between the physicochemical properties of catalysts and the catalytic activity were revealed.

## Data Availability

The authors of this paper also conducted similar experiments and extracted different data sets. The raw data supporting the conclusions of this article will be madeavailable by the authors upon request.

## Supplementary Materials

The following supporting information can be downloaded at: https://www.mdpi.com/article/10.3390/ijms252111582/s1. References [12,15,26,37,38,39,40,41,42] are cited in the supplementary materials.
