# Supplementary material for: Peroxymonosulfate Activation by Rice-Husk-Derived Biochar (RBC) for the Degradation of Sulfamethoxazole: The Key Role of Hydroxyl Groups
Source: Int J Mol Sci. 2024 Oct 29;25(21):11582. doi: 10.3390/ijms252111582 (PMC11545899; doi:10.3390/ijms252111582)
Supplement: Supplementary file 1 [file ijms-25-11582-s001.zip › ijms-3130760-supplementary.pdf]

Supplementary Material

Reagents and chemicals

The chemical reagents employed in this work include sulfamethoxazole (SMX, 98.0%, Rhawn), cephalexin (CEX, 99.7%, Rhawn), ciprofloxacin (CIP, 98.0%, Rhawn), 4-chloro-3-methyl phenol (CMP, 99.0%, Rhawn), Triclosan (TCS, 97.0%, Rhawn), sodium thiosulfate ($Na_2S_2O_3$, 99.0%, Rhawn), *tert*-Butanol (TBA, ≥99.5%, Rhawn), methanol (MeOH, ≥99.5%, Rhawn), and *p*-Benzoquinone (*p*-BQ, ≥99.0%, Rhawn), furfuryl alcohol (FFA, ≥98.0%, Aladdin), hydrochloric acid (HCl, 36.0−38.0%, Rhawn), sulfuric acid (H_2SO_4, 98.0%, Rhawn), sodium hydroxide (NaOH, 97.0%, Rhawn), sodium chloride (NaCl, 99.5%, Aladdin), monopotassium phosphate (KH_2PO_4, 99.0%, Aladdin), sodium bicarbonate ($NaHCO_3$, 99.8%, Aladdin), humic acid (HA, 98.0%, Aladdin), peroxymonosulfate ($KHSO_5 \cdot 0.5KHSO_4 \cdot 0.5K_2SO_4$, Oxone, Aladdin), 5,5-Dimethyl-1-pyrroline-N-oxide (DMPO, 97.0%, Rhawn), 2,2,6,6-tetramethyl-4-piperidinol (TEMP, ≥98.0%, Aladdin). Real water samples including tap water, and river water, were collected from the municipal water company, and Nanfei River in Anhui Province, China. All chemicals were employed without further purification and deionized water (18.2 MΩ/cm) was used throughout the experiments.

Characterization and analysis methods

Morphologies of RBC_{800} were observed by the scanning electron microscope (SEM, Hitachi SU8020) and high-resolution transmission electron microscope (HR-TEM, JEOL JEM-2100). The crystalline structure of RBC_{800} was detected by the X-ray diffractometer (PANalytical, Netherlands). ATR-FTIR spectra were obtained by a FT-IR spectrometer (Thermo, Nicolet 6700) in the 4,000−500 cm^{-1} range. The chemical states of RBC_{800} were analyzed by X-ray photoelectron spectroscopy (XPS, Thermo, ESCALAB250Xi). The SSAs and pore size distributions were analyzed with the Brunauer-Emmett-Teller (BET) method (Autosorb-IQ3, Quantachrome, USA). Raman spectrometer (HORIBA JOBIN YVON, LabRAM HR Evolution) was used to acquire Raman spectra.

SMX concentration was determined by high-performance liquid chromatography (HPLC, Shimadzu, LC-20AT). The mobile phase was amixture of acetonitrile/deionized water (0.1% formic acid) (70%/30%, v/v) at a flow-rate of 1.0 $mL{\cdot}min^{-1}$. The wavelength and column temperature were set to 255 nm and 30°C, respectively. The decomposition rate of PMS was determined by an ABTS colorimetric method (Wu et al., 2020). Total organic carbon (TOC) was measured using a Shimadzu TOC-vcph analyzer (Multi N/C 3000). The zeta potential of RBC_{800} was determined with a Zetasizer NanoBrook Omni (Brookhaven). Radical studies were performed with a JES-FA200 (JEOL) spectrometer. The intermediates were detected with ultra-performance liquid chromatography to quadrupole time-of-flight mass spectrometry

(UPLC-TOF/MS, ACQUITY UPLC LCT Premier XE, America).

Theoretical calculations

All DFT calculations are performed in Gaussian 16 software, using B3LYP/6-31 G (d, p) basis sets for optimization and transition state search for all configurations. All configuration optimization is carried out without symmetry restriction, and vibration analysis is performed after optimization to ensure no phantom frequency. Vibration analysis is performed on all transition state structures to ensure that there is only one virtual frequency, and that the virtual frequency corresponds to the direction of vibration to connect the reactants and products. The adsorption energy of AB structure is calculated based on the formula $E_b = E(AB) - E(A) - E(B)$, and the Gibbs free energy variation (ΔG) is also obtained under the method B3LYP/6-31G (d, p).

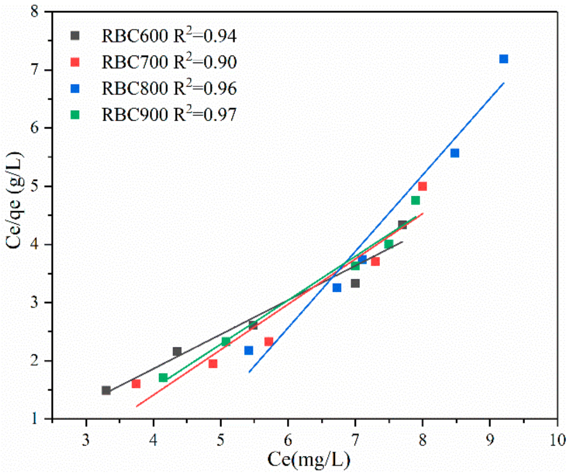

Fig. S1. Langmuir isotherm plots for the sorption of SMX on catalysts (Conditions: pH_0 = 7.0, $[SMX]_0$ = 10.0 mg/L, $[RBC_{800}]$ = 0.4 g/L, $[PMS]_0$ = 0.6 mM, reaction time = 200 min, T = 25°C).

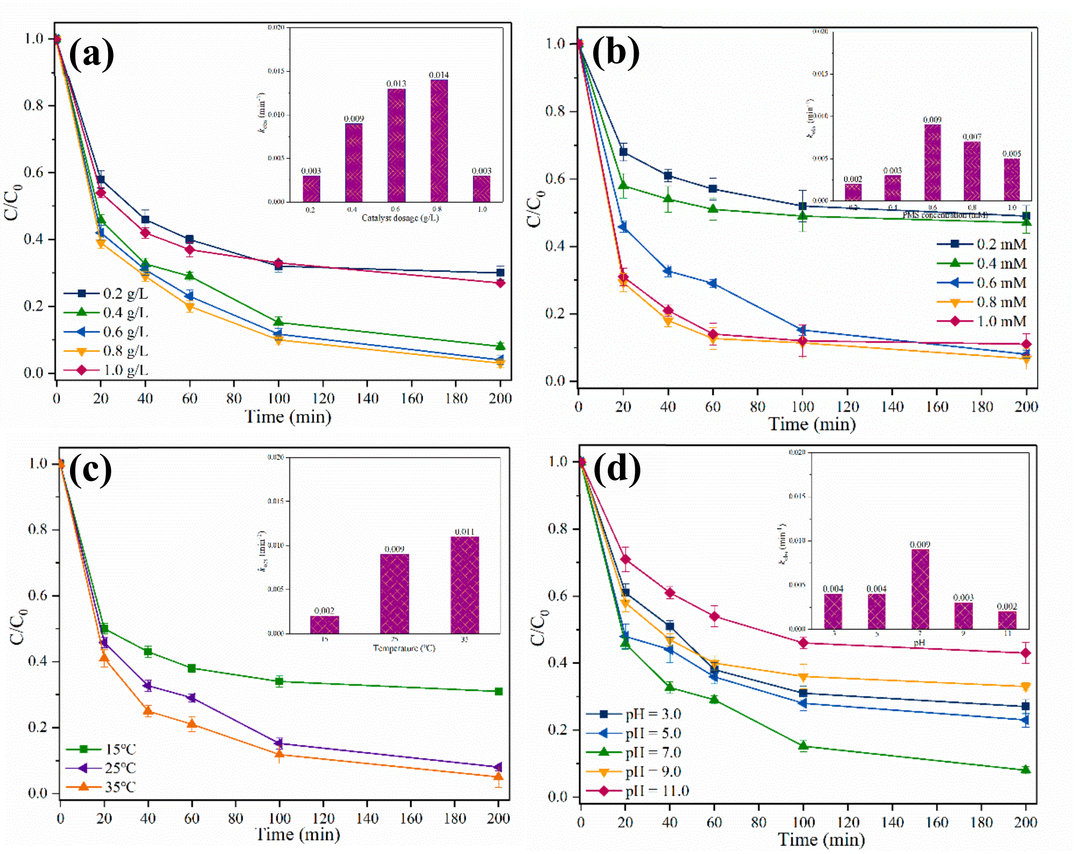

Fig. S2. Effects of various parameters on SMX degradation: (a) RBC_{800} dosage, (b) PMS concentration, (c) temperature, and (d) pH (Conditions: $pH_0 = 7.0$, $[SMX]_0 = 10.0$ mg/L, $[RBC_{800}] = 0.4$ g/L, $[PMS]_0 = 0.6$ mM, reaction time = 200 min, T = 25℃).

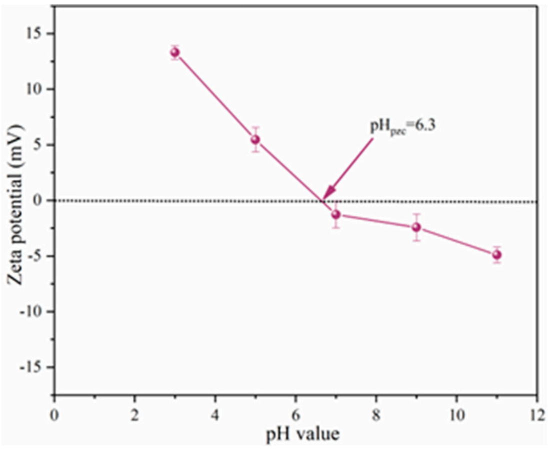

Fig. S3. Zeta potentials of RBC_{800}

Effects of RBC_{800} dosage. Different concentrations of RBC_{800} (0.2, 0.4, 0.6, 0.8 and 1.0 g/L) effect on the SMX degradation rate is displayed in Fig. S2(a). Apparently, the SMX removal rate increased from 70.0% to 97.0% with the amount of RBC_{800} increased from 0.2 g/L to 0.8 g/L, which was due to more active sites for PMS activation. The corresponding k_{obs} increased from 0.003 min^{-1} to 0.014 min^{-1}. However, the SMX removal rate decreased to 73.0% within 200 min when the RBC_{800} dosage was 1.0 g/L in the RBC_{800}/PMS system. Excessive RBC_{800} dosage (1.0 g/L) may result in the quenching of partial active species (Eqs. (1−3)) (Zhao et al., 2020).

$$\cdot OH + \cdot OH \rightarrow H_2O_2 \quad (1)$$

$$SO_4^{\cdot-} + SO_4^{\cdot-} \rightarrow S_2O_8^{2-} \quad (2)$$

$$\cdot OH + SO_4^{\cdot-} \rightarrow HSO_5^{-} \quad (3)$$

Effects of PMS concentration. The effect of PMS dosage on SMX elimination is shown in Fig. S2(b). The degradation rate was 51.0%, 53.0%, 92.0% and 93.3% when the PMS dosage was 0.2, 0.4, 0.6 and 0.8 mM, respectively. When PMS dosage was further increased from 0.8 mM to 1.0 mM, the removal rate and k_{obs} slightly decreased to 89.0% and 0.005 min^{-1}, which may be due to the quenching of ROSs caused by

excessive PMS dosage (Eqs. (4−5)) (Wang and Chen, 2022).

$$HSO_5^- + SO_4^{\cdot-} \rightarrow SO_4^{2-} + SO_5^{\cdot-} + H^+ \tag{4}$$

$$HSO_5^- + \cdot OH \rightarrow SO_5^{\cdot-} + H_2O \tag{5}$$

Effects of temperature. The removal rate of SMX in the RBC_{800}/PMS system at 15, 25, and 35°C is depicted in Fig. S2(c). The degradation rate of SMX increased rapidly from 69.0% to 95.0% with the increasing of temperature (15−35°C). These results reveal that a increasing temperature is able to promote the removal of SMX and accelerate the decomposition of PMS.

Effects of solution pH. The effect of solution pH on the SMX removal is examined (Fig. S2(d)). The elimination rate of SMX was 73.0%, 77.0%, 92.0%, 67.0%, and 57.0% within 200 min in the RBC_{800}/PMS system at pH 3.0, 5.0, 7.0, 9.0, and 11.0, respectively. Obviously, the neutral condition (pH = 7.0) is favorable for the removal of SMX. H_2SO_5 replaced HSO_5^- and participate in the degradation process at acidic conditions. Besides, lots of H^+ in the reaction solution could interact with reactive species, which had an adverse effect on the SMX elimination (Eqs. (6−7)). As displayed in Fig. S3, the point of zero charge (pH_{pzc}) of RBC_{800} was 6.3, suggesting that the surface of RBC_{800} was negatively charged at basic conditions. The electrostatic repulsion effect caused the barrier between HSO_5^- and RBC_{800}, and thus hindered the generation of active species (Zhang et al., 2020).

$$\cdot OH + H^+ + e^- \rightarrow H_2O \tag{6}$$

$$SO_4^{\cdot-} + H^+ + e^- \rightarrow HSO_4^{\cdot-} \tag{7}$$

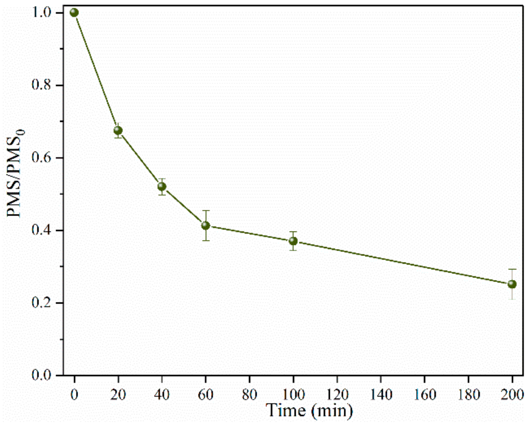

Fig. S4. Residual PMS in the RBC_{800}/PMS system (Conditions: $pH_0 = 7.0$, $[SMX]_0 = 10.0$ mg/L, $[RBC_{800}] = 0.4$ g/L, $[PMS]_0 = 0.6$ mM, reaction time = 200 min, T = 25℃).

Residual PMS. To explore the catalytic performance of RBC_{800} in depth, the residual PMS concentration was detected by the ABTs colorimetric method. The decomposition rate of PMS in the RBC_{800}/PMS system was 74.9% within 200 min (Fig. S4). It was suggested that the favorable degradation rate of SMX in the RBC_{800}/PMS system could be due to the rapid decomposition of PMS.

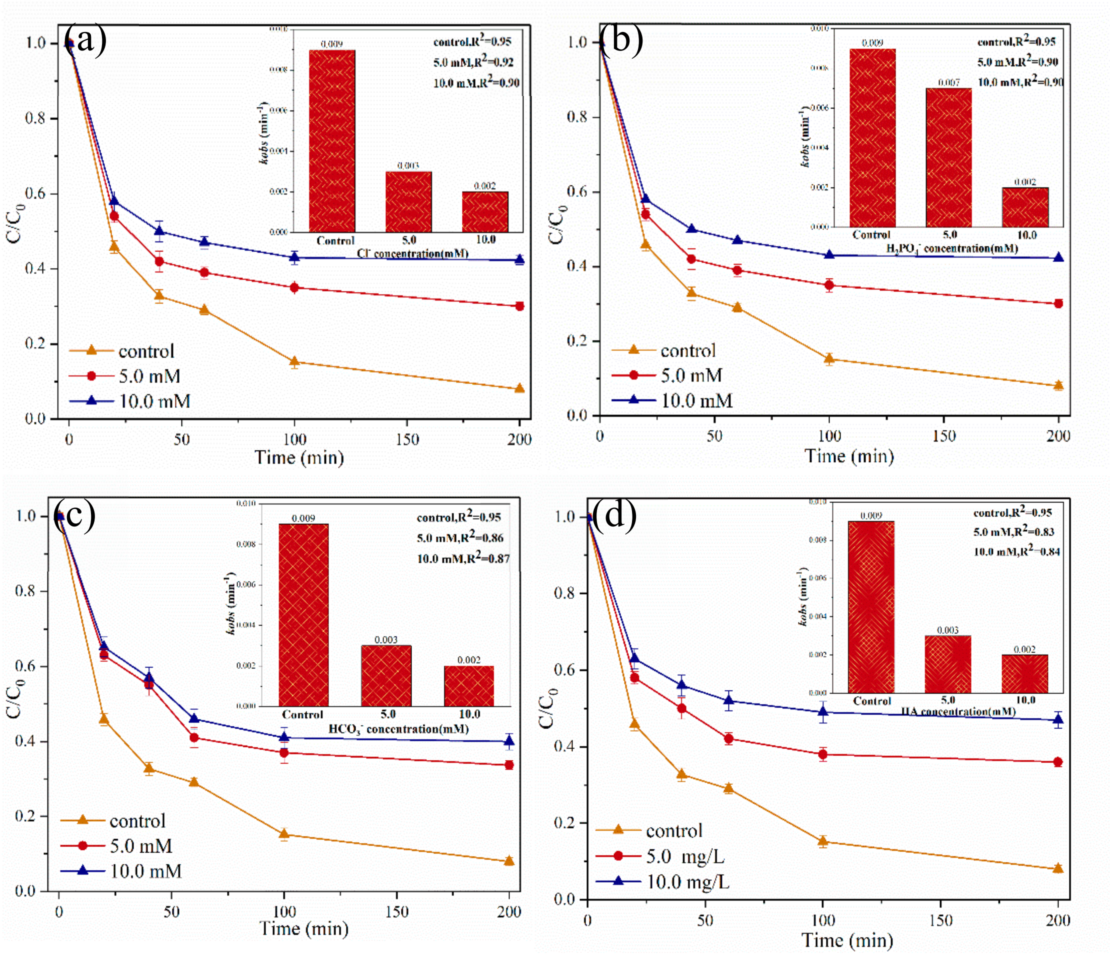

Fig. S5. Effects of Cl^- (a), $H_2PO_4^-$ (b), HCO_3^- (c) and HA (d) on SMX degradation. (Conditions: $[SMX]_0 = 10.0$ mg/L, $[RBC_{800}] = 0.4$ g/L, $[PMS]_0 = 0.6$ mM, $pH_0 = 7.0$, T = 25℃).

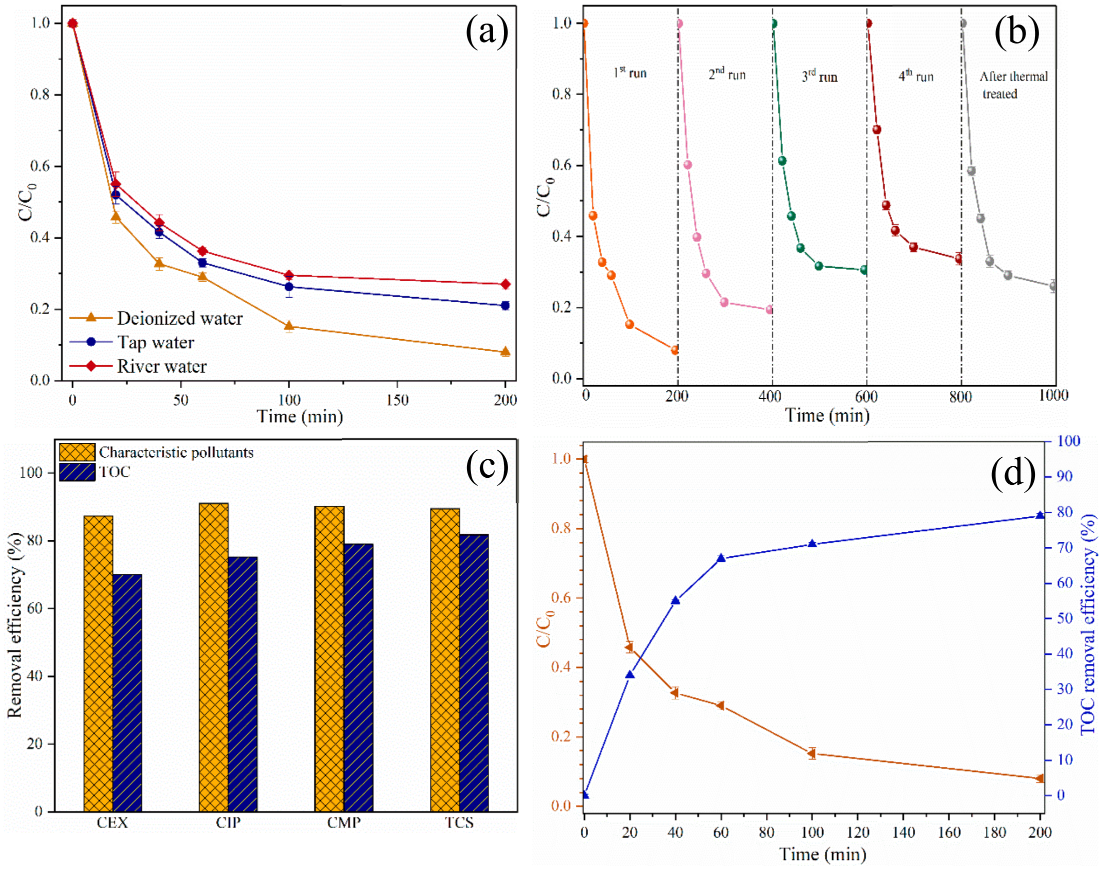

Fig. S6. (a) SMX removal in different water matrices, (b) reusability test, (c) the removal of various PPCPs in RBC_{800}/PMS system, and (d) mineralization efficiencies of SMX (Conditions: $[SMX]_0 = 10.0$ mg/L, $[RBC_{800}] = 0.4$ g/L, $[PMS]_0 = 0.6$ mM, $pH_0 = 7.0$, T = 25°C, reaction time = 200 min).

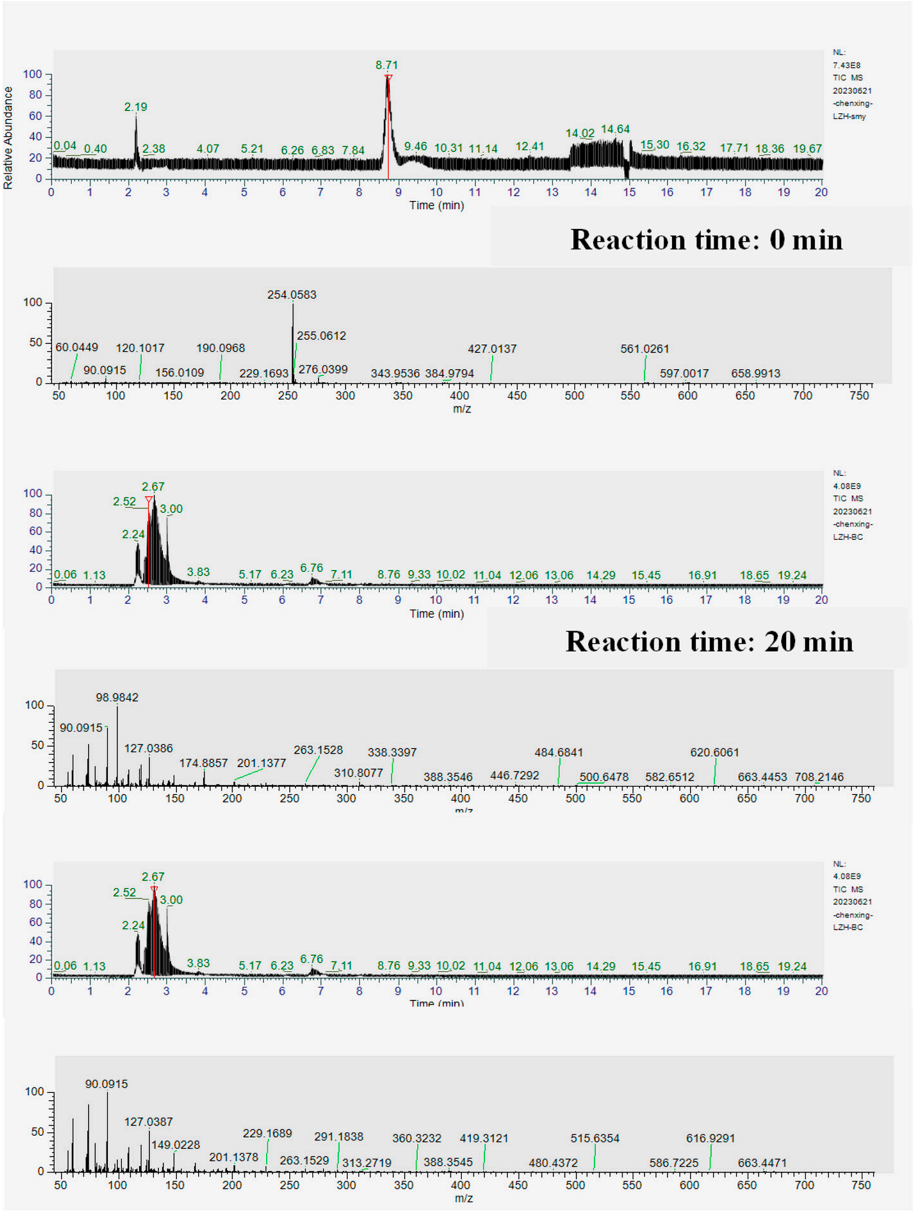

Fig. S7. Mass spectra of intermediate products.

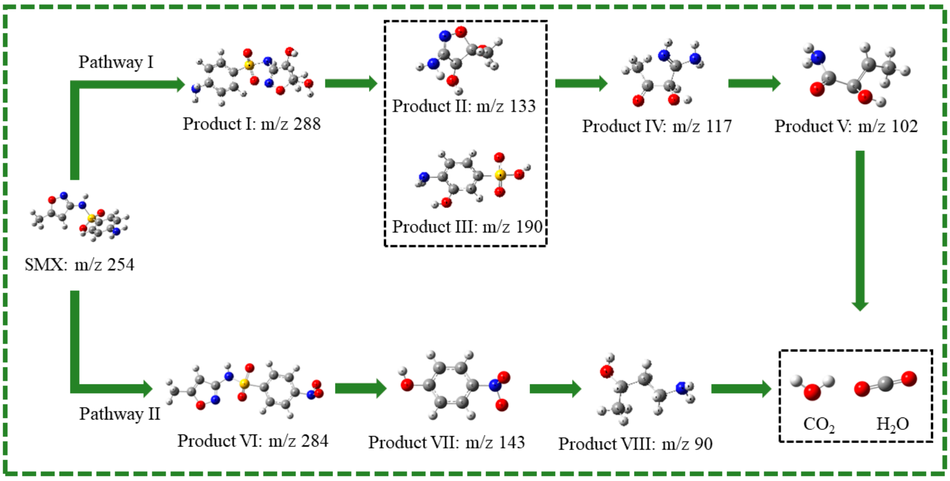

Fig. S8. Possible pathways for degradation of SMX in the RBC_{800}/PMS system.

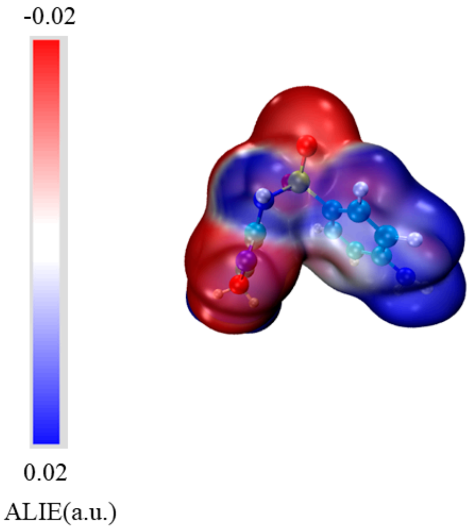

Fig. S9. Charge distribution of SMX.

Table S1. Basic information of prepared catalysts.

Samples	S_{BET} (m^2/g) $_a$	Pore volume (cm^3/g) $_a$	Average pore diameter (nm) $_a$	I_D/I_G $_b$
RBC_{600}	65.00	0.054	3.32	-
RBC_{700}	124.11	0.096	2.67	0.98
RBC_{800}	194.86	0.111	2.46	1.07
RBC_{900}	102.71	0.078	2.59	0.88

[a] Obtained by Langmuir modelling studies.

[b] Analyzed by Raman spectra.

Table S2. Comparison of the k_{obs} and R^2 of different systems.

Systems	k_{obs} (min^{-1})	R^2
RBC_{600}+PMS	0.006	0.95
RBC_{700}+PMS	0.007	0.95
RBC_{800}+PMS	0.009	0.95
RBC_{900}+PMS	0.002	0.80

Table S3. Studies of the PPCPs degradation with PS activated by biochar-based catalysts.

PPCPs	Catalyst	Oxidant source	Performance
0.05 mM	150 mg/L CSBC	0.05 mM PMS	The SMX removal rate was 85.0% within 30 min at pH 5.0 in the presence of 150 mg/L of CSBC (Hung et al., 2022).
10.0 mg/L	0.5 g/L PC700	0.8 mM PS	The PC700/PS system removed 68.1% of SMX. Reaction conditions: PS: 2 mmol/L, Fe^{3+}: 1 mmol/L, pH = 3.5, temperature: 25°C, SMX: 10 mg/L, PC700: 0.5 g/L (Liang et al., 2021).
5.0 mg/L	0.05 g/L EGB-900	4.0 mM PS	SMX can be completely removed within 90 min at EGB-900 concentration of 0.05 g/L and the k_{obs} value was 0.0655 min^{-1} (Qi et al., 2020).
100.0 mg/L	0.2 g/L biochar	0.5 g/L PDS	After the addition of 0.5 g/L PDS, over 98% removal efficiency of 2,4-DCP could be reached in the following 60 min (Yu et al., 2020).
0.034 mM	1.0 g/L SBC	0.8 mM PMS	TCS removal efficiency achieved 66.7%. Reaction conditions: PMS: 0.8 mmol/L, pH = 7.2, temperature: 25°C, TCS: 0.034 mM, SBC: 1.0 g/L (Wang and Wang, 2019).

Table S4. Characteristics of different water matrices.

	Deionized water	Tap water	River water
pH	6.8	7.2	7.8
TOC (mg/L)	-	2.8	7.9
DO (mg/L)	8.1	7.1	4.8
SS (mg/L)	-	-	45.9
Cl^- (mg/L)	-	4.8	14.8
SO_4^{2-} (mg/L)	-	-	12.9
NO_3^- (mg/L)	-	-	6.8
UV_{254}	-	0.04	0.03

Table S5. The relevant parameters of different configurations.

Configurations	D (Å)	$l_{o\text{-}o}$ (Å)	Transition state energy barrier (kcal/mol)
C/PMS	2.18	1.454	
C/PMS TS	2.17	1.972	35.16
COOH/PMS	1.46	1.463	
COOH/PMS TS	1.77	2.187	31.93
C=O/PMS	2.01	1.458	
C=O/PMS TS	1.89	2.120	32.28
C-OH/PMS	1.43	1.468	
C-OH/PMS TS	1.71	2.296	28.12

Table S6. Intermediates of SMX degradation detected by UPLC-TOF/MS.

Structural formula	Molecular formula	m/z
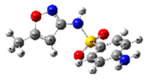	$C_{10}H_{11}N_3O_3S$	254
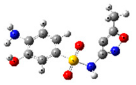	$C_{10}H_{10}N_3O_4S$	270
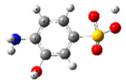	$C_6H_7NO_4S$	190
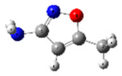	$C_4H_6N_2O$	99
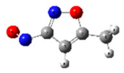	$C_4H_4N_2O_2$	111
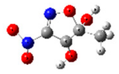	$C_4H_5N_2O_5$	133
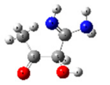	$C_4H_8N_2O_2$	117
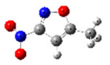	$C_4H_4N_2O_3$	127

References

Hung, C.M., Chen, C.W., Huang, C.P., Shiung Lam, S., Dong, C.D., 2022. Peroxymonosulfate activation by a metal-free biochar for sulfonamide antibiotic removal in water and associated bacterial community composition. Bioresour Technol 343, 126082.

Liang, J., Duan, X., Xu, X., Chen, K., Wu, F., Qiu, H., Liu, C., Wang, S., Cao, X., 2021. Biomass-derived pyrolytic carbons accelerated Fe(III)/Fe(II) redox cycle for persulfate activation: Pyrolysis temperature-depended performance and mechanisms. Applied Catalysis B: Environmental 297.

Qi, Y., Ge, B., Zhang, Y., Jiang, B., Wang, C., Akram, M., Xu, X., 2020. Three-dimensional porous graphene-like biochar derived from Enteromorpha as a persulfate activator for sulfamethoxazole degradation: Role of graphitic N and radicals transformation. J Hazard Mater 399, 123039.

Wang, S., Wang, J., 2019. Activation of peroxymonosulfate by sludge-derived biochar for the degradation of triclosan in water and wastewater. Chemical Engineering Journal 356, 350-358.

Wang, W., Chen, M., 2022. Catalytic degradation of sulfamethoxazole by peroxymonosulfate activation system composed of nitrogen-doped biochar from pomelo peel: Important roles of defects and nitrogen, and detoxification of intermediates. J Colloid Interface Sci 613, 57-70.

Wu, Z., Wang, Y., Xiong, Z., Ao, Z., Pu, S., Yao, G., Lai, B., 2020. Core-shell magnetic Fe3O4@Zn/Co-ZIFs to activate peroxymonosulfate for highly efficient degradation of carbamazepine. Applied Catalysis B: Environmental 277.

Yu, J., Tang, L., Pang, Y., Zeng, G., Feng, H., Zou, J., Wang, J., Feng, C., Zhu, X., Ouyang, X., Tan, J., 2020. Hierarchical porous biochar from shrimp shell for persulfate activation: A two-electron transfer path and key impact factors. Applied Catalysis B: Environmental 260.

Zhang, H., Song, Y., Nengzi, L.-c., Gou, J., Li, B., Cheng, X., 2020. Activation of persulfate by a novel magnetic CuFe2O4/Bi2O3 composite for lomefloxacin degradation. Chemical Engineering Journal 379.

Zhao, Y., Song, M., Cao, Q., Sun, P., Chen, Y., Meng, F., 2020. The superoxide radicals' production via persulfate activated with CuFe2O4@Biochar composites to promote the redox pairs cycling for efficient degradation of o-nitrochlorobenzene in soil. J Hazard Mater 400, 122887.
